# Evaluation of RMES, an Automated Software Tool Utilizing AI, for Literature Screening with Reference to Published Systematic Reviews as Case-Studies: Development and Usability Study

**DOI:** 10.2196/55827

**Published:** 2024-12-09

**Authors:** Ayaka Sugiura, Satoshi Saegusa, Yingzi Jin, Riki Yoshimoto, Nicholas D Smith, Koji Dohi, Tadashi Higuchi, Tomotake Kozu

**Affiliations:** 1 Deloitte Analytics Deloitte Tohmatsu Risk Advisory LLC Tokyo Japan; 2 Evidence Generation ＆ Communication Division EMC K.K. Tokyo Japan

**Keywords:** artificial intelligence, automated literature screening, natural language processing, randomized controlled trials, Rapid Medical Evidence Synthesis, RMES, systematic reviews, text mining

## Abstract

**Background:**

Systematic reviews and meta-analyses are important to evidence-based medicine, but the information retrieval and literature screening procedures are burdensome tasks. Rapid Medical Evidence Synthesis (RMES; Deloitte Tohmatsu Risk Advisory LLC) is a software designed to support information retrieval, literature screening, and data extraction for evidence-based medicine.

**Objective:**

This study aimed to evaluate the accuracy of RMES for literature screening with reference to published systematic reviews.

**Methods:**

We used RMES to automatically screen the titles and abstracts of PubMed-indexed articles included in 12 systematic reviews across 6 medical fields, by applying 4 filters: (1) study type; (2) study type + disease; (3) study type + intervention; and (4) study type + disease + intervention. We determined the numbers of articles correctly included by each filter relative to those included by the authors of each systematic review. Only PubMed-indexed articles were assessed.

**Results:**

Across the 12 reviews, the number of articles analyzed by RMES ranged from 46 to 5612. The number of PubMed-cited articles included in the reviews ranged from 4 to 47. The median (range) percentage of articles correctly labeled by RMES using filters 1-4 were: 80.9% (57.1%-100%), 65.2% (34.1%-81.8%), 70.5% (0%-100%), and 58.6% (0%-81.8%), respectively.

**Conclusions:**

This study demonstrated good performance and accuracy of RMES for the initial screening of the titles and abstracts of articles for use in systematic reviews. RMES has the potential to reduce the workload involved in the initial screening of published studies.

## Introduction

Systematic reviews are informative but labor-intensive, requiring significant workload to search for and screen articles from literature reviews. Rapid Medical Evidence Synthesis (RMES; Deloitte Tohmatsu Risk Advisory LLC) is a semiautomated, artificial intelligence (AI)–based software tool that was developed to facilitate screening of large numbers of articles for systematic reviews, for example [1]. In this study, we tested the application of RMES for screening articles with reference to published systematic reviews.

Randomized controlled trials (RCTs) are a fundamental component of drug development, guideline development, and medical decision-making. Multiple RCTs may be necessary to confirm previous findings or explore the effectiveness and safety in different patient populations to account for differences in disease stage, concomitant therapies, and ethnicities. This means stakeholders may need to consider the results of multiple clinical trials in order to make robust conclusions to support evidence-based medicine (EBM) [2], through systematic reviews, meta-analyses, and scoping reviews. These reports may be used to evaluate the available evidence before conducting additional studies, to support the development of clinical guidelines or recommendations, or to support new drug applications or a change in the approved label, for example.

After defining the research question, the author must perform literature searches of an appropriate database (eg, PubMed) for relevant articles. For EBM, this often involves searches for RCTs comparing the chosen intervention with a comparator (eg, placebo, active treatment, or control treatment). However, this poses a challenge to the author; a simple search of PubMed using the term “randomized controlled trial” and the filter “Clinical Trial” yielded over 600,000 published articles since 1960, of which 250,000 were published in the last 10 years. Therefore, depending on the chosen setting and objective, the initial literature search can yield 100s or even 10,000s of titles and abstracts that would need to be screened.

EBM is often outdated before it is even published [2] because the initial literature screening imposes a huge burden in terms of the time required to screen the articles, the time needed to extract and analyze the data, and the time taken to publish the findings. Therefore, there is a clear need for developing semiautomated literature screening software tools that can reduce the burden associated with initial screening. Such tools may also facilitate the process of identifying RCTs published since the initial literature searches.

The last decade has seen great strides in the development of AI, deep learning, and natural language processing (NLP), and they are now being adapted for automated literature screening [3-21]. Some examples include Elicit [22], which is used for reference retrieval and organization, and can be used for data extraction. AUTOMETA is a program that can identify and extract Participants, Intervention, Control, and Outcome (PICO) elements [14,15]. DistillerSR [10,23] learns the inclusion and exclusion criteria from a small training set and can be used to perform literature searches, screen articles, and record data extracted from the articles. In addition, the Cochrane Center has introduced Screen4Me, which involves a combination of machine learning and manual review (through crowdsourcing) for classifying articles as RCTs or not [16,24].

**Figure 1:**
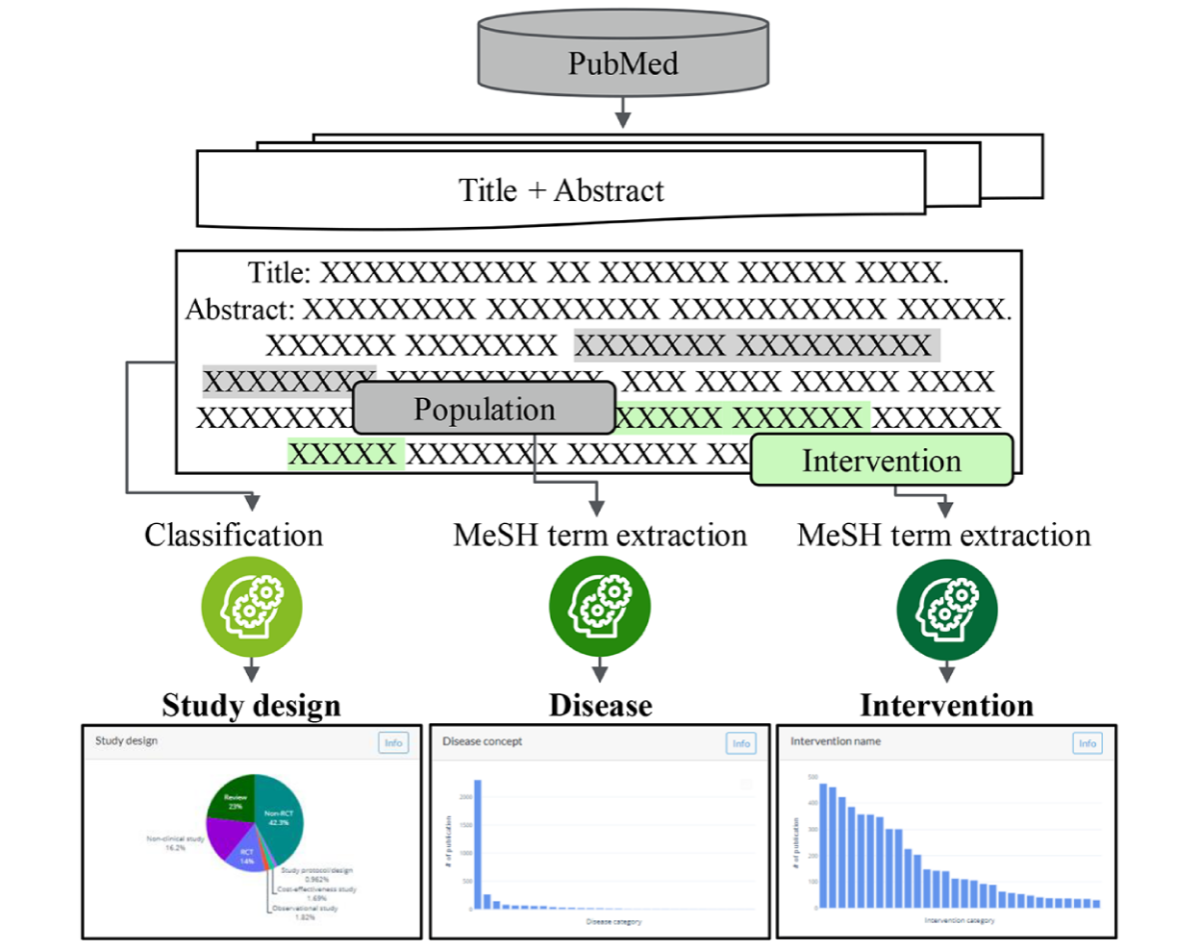
Schematic of the Rapid Medical Evidence Synthesis (RMES) processes. MeSH: Medical Subject Headings.

RMES is a software tool that was developed to support information retrieval, data extraction, and data analysis for use in EBM, such as systematic reviews and meta-analysis. The design of RMES was recently described [1]. One component of the software is that it is capable of performing automated screening of the titles and abstracts of published work that uses the PICO format. This involves a PubMedBERT model trained on weighted EBM-NLP and weighted automatically labeled data [25]. PubMedBERT was tuned using data retrieved from ClinicalTrials.gov through its application programming interface [26] and a dataset comprising 1807 articles with a title, abstract, and NCT identifier that could be linked to the ClinicalTrials.gov data. RMES can determine the study design and categorize the disease and interventions according to Medical Subject Headings (MeSH) using MetaMap (Incode Technologies) [27], and hence narrow down the studies relevant to the research topic without placing any additional workload on the researchers (Figure 1).

RMES is able to identify the study type, diseases, and interventions from the title and abstract automatically, and these can be used in any combination as filters to screen the articles. The study type filter can be used to classify articles based on study designs (eg, RCT). The disease and intervention are categorized into terms within MeSH. When applying disease or intervention filters, the researcher does not need to manually input the disease or intervention name, because RMES identifies the diseases described in the article extracted from the PubMed search and displays the 30 most commonly mentioned categories. The user can then select the disease or intervention of interest and filter the articles automatically.

While the development and performance of the internal algorithms used in RMES were described [1], its accuracy for automated screening of titles and abstracts in the context of literature searches for systematic reviews has not been reported. Therefore, our objective in this study was to evaluate its accuracy for selecting articles by comparing it to the articles screened and included in 12 published, peer-reviewed systematic reviews of RCTs across 6 different medical fields. Because RMES is currently only able to screen articles indexed in PubMed, its performance was assessed in terms of the accuracy for excluding unnecessary or irrelevant articles with reference to PubMed-indexed articles that were screened and included in the published reviews.

## Methods

### Evaluation of RMES

In this study, we used RMES (version r0.16.2) for literature screening. To evaluate the accuracy of RMES, we chose 12 published systematic reviews that satisfied the eligibility criteria shown in Textbox 1.

We selected 6 broad medical fields to test the generalizability of RMES to different settings, such as malignant neoplasms [28,29], heart disease [30,31], cerebrovascular disease [32,33], hypertension [34,35], diabetes [36,37], and dietary supplements [38,39]. We selected these fields because malignant neoplasms, heart disease, and cerebrovascular disease are the 3 leading causes of death, and hypertension and diabetes are 2 of the most common chronic diseases in Japan [40,41]. We included dietary supplements, especially multivitamin mineral, as the sixth field because of their high frequency of use among adults [42]. Table S1 in Multimedia Appendix 1 shows the search queries used to retrieve systematic reviews in each field, the number of articles identified by the searches, the number of articles excluded because they did not satisfy the criteria above, and the total number of potential candidates. We selected the 2 most recently published systematic reviews in each medical field, yielding a total of 12 analyzed systematic reviews.

**Eligibility criteria.**

**Inclusion criteria**

- Systematic reviews (SRs) published in the Cochrane Database of Systematic reviews.
- SRs that were published as open access.
- SRs that used randomized controlled trials as the data source.
- SRs that reported all the references used by the authors.
- SRs that assessed the efficacy of a drug or dietary supplement.

For each systematic review, we recreated the search query for PubMed or MEDLINE (Table S2 in Multimedia Appendix 1) to retrieve published articles, which were then imported into RMES. We also labeled any articles that were not indexed in PubMed or articles that lacked an abstract; these articles could not be screened using RMES and were therefore excluded for the accuracy of analysis. For each systematic review, the PubMed-indexed articles were entered into RMES and filtered using 4 combinations of filters: (1) study type, (2) study type + disease, (3) study type + intervention, and (4) study type + disease + intervention (Table S3 in Multimedia Appendix 1) and labeled for inclusion or exclusion.

Here, we evaluated the performance of RMES by comparing the numbers of articles the software excluded by applying each of the 4 filters with the articles extracted from PubMed for the 12 systematic reviews.

### Statistical Analyses

Data were analyzed descriptively in terms of the number and percentage of PubMed-indexed articles that were included and excluded by the 4 RMES filters, as follows. We calculated the number of articles included in the systematic reviews minus the number of articles included in the systematic review that were not excluded by each RMES filter. We also determined the number of articles excluded by RMES as the number of articles retrieved from PubMed that were imported to RMES minus the number of articles that were not excluded by the RMES filter. These outcomes are defined in Table 1. These analyses were performed for all 4 filters for all 12 systematic reviews. Only PubMed-indexed articles included or excluded in each systematic review were analyzed. Data analyses were conducted using Microsoft Excel.

**Table 1:** Definitions of search and performance parameters.

Parameter			Definition
**Search parameters**			
	#1. Number of PubMed-indexed articles retrieved in the SR^a^	Total number of articles listed in the PubMed database that were retrieved by the authors of the SR using their literature search.	
	#2. Source of articles ultimately used in the SR	Source and number of articles that satisfied the authors’ eligibility criteria and subjected to data extraction. For this study, the articles were classified by the source depending on whether the articles were originally retrieved from “any source” (eg, PubMed, Embase, other databases, or manual review of published articles) or from “PubMed.” We used the number of PubMed-indexed articles as the denominator to calculate percentages of articles included in the SR.	
	#3. Number of articles retrieved from PubMed by our search	The number of articles that we retrieved after performing the modified search queries for each SR. Because it was not possible to perfectly replicate the authors’ original searches, the number of articles retrieved from our searches may differ from the number of PubMed-indexed articles retrieved by the authors of the SR (#1). Our modified searches are listed in Table S2 in Multimedia Appendix 1.	
	#4. Number of PubMed-cited articles included in the SR retrieved by our search	The number of articles retrieved by our search of PubMed that were included in the authors’ SR. The percentage was calculated using #2 as the denominator. A value of 100% indicates that our search retrieved all PubMed-cited articles that were used by the authors in their SR.	
	#5. Total number of articles available for analysis using RMES^b^	The total number of PubMed-cited articles retrieved by our search that had an abstract and could be analyzed using RMES, as a subset of #3.	
	#6. Number of articles included in the SR	The number of PubMed-cited articles that were included in the SR. The percentage was calculated using #2 as the denominator.	
**Performance parameters**			
	#7. Articles assessed using RMES (*a*)	The number of PubMed-cited articles with an abstract retrieved by our literature search and evaluated by RMES (#5).	
	#8. Articles included in SR (*b*)	The number of PubMed-cited articles used by the authors in their SR (#2).	
	#9. Articles assessed using RMES (*a*)	The number of articles analyzed using RMES (#5).	
	#10. Articles excluded by the filter (*c*)	The number of PubMed-cited articles with an abstract analyzed using RMES and subsequently excluded after applying the indicated filter, as a subset of #9.	
	#11. Articles selected by the filter (for manual screening)	The number of PubMed-cited articles with an abstract analyzed using RMES and retained by the indicated filter, as a subset of #9. These articles would be eligible for manual screening.	
	#12. Reduction rate (%) (*=c/a*)	The percentage of articles excluded by the indicated RMES filter, calculated as the number of articles excluded (#10) divided by the number of articles assessed (#9). A value of 0% indicates that the RMES filter retained all of the PubMed-cited articles with an abstract that were used by the authors in their SR. A value of 100% indicates that the RMES filter incorrectly excluded all of those articles.	
	#13. Articles incorrectly excluded by the filter (*d*)	The number of articles that were excluded by the indicated filter, but were included by the authors in their SR (as defined in #2).	
	#14. Selection success rate (%) (*=[b−d]/b*)	The percentage of articles that were retained by the indicated RMES filter out of the total number of eligible articles. The denominator was the number of PubMed-cited articles included in the SR by the authors (as defined in #2). A value of 100% indicates that the RMES filter correctly identified all of the PubMed-cited articles with an abstract that were used by the authors in their SR. A value of 0% indicates that the RMES filter did not retain any of those articles.	

^a^SR: systematic review.

^b^RMES: Rapid Medical Evidence Synthesis.

### Ethical Considerations

Ethical review was not required for this study owing to the use of data from published studies.

## Results

### Characteristics of the Systematic Reviews

The 12 systematic reviews analyzed in this study are summarized in Table 2. All of the systematic reviews examined RCTs. Most of the studies involved a single type of comparator (eg, placebo or a conventional control treatment), but some included multiple comparators. For example, Ferrara et al [29] compared first-line immune checkpoint inhibitor therapy as monotherapy or in combination versus platinum-based chemotherapy, with or without bevacizumab, in patients with advanced non-small cell lung cancer. Hemmingsen et al [36] compared (ultra-)long-acting insulin analogs versus neutral protamine Hagedorn insulin or other (ultra-)long-acting insulin analogs in patients with type 1 diabetes mellitus. Gnesin et al [37] compared metformin monotherapy versus no intervention, behavior-changing interventions, or other glucose-lowering drugs in patients with type 2 diabetes mellitus.

Table 3 shows the numbers of articles that were screened by the authors in each systematic review, which ranged from 37 in the review by Burckhardt et al [38] to 4935 in the review by Ferrara et al [29]. The number of articles ultimately included in the systematic reviews ranged from 7 to 202 regardless of the original source (PubMed or other sources) and from 4 to 53 for articles indexed in PubMed.

**Table 2:** Overview of the systematic reviews^a^.

General field and citation			Setting and disease		Intervention
**Malignant neoplasm**					
	Zengerling et al [28]	Men with advanced hormone-sensitive prostate cancer		Degarelix versus standard androgen suppression therapy	
	Ferrara et al [29]	Advanced non-small cell lung cancer		First-line immune checkpoint inhibitor as monotherapy or in combination versus platinum-based chemotherapy, with or without bevacizumab	
**Heart disease**					
	Safi et al [30]	People without heart failure and with left ventricular ejection fraction >40% in the nonacute phase after myocardial infarction		β-blockers versus placebo or no treatment	
	Sethi et al [31]	Secondary prevention of coronary heart disease		Antibiotics versus placebo or no treatment	
**Cerebrovascular disease**					
	Roaldsen et al [32]	Acute ischemic stroke presenting on awakening from sleep		Intravenous thrombolysis and endovascular thrombectomy versus control treatment	
	Martí-Carvajal et al [33]	Acute ischemic stroke		Citicoline versus placebo	
**Hypertension**					
	Wang et al [34]	Primary hypertension		Renin inhibitors versus angiotensin converting enzyme inhibitors	
	Musini et al [35]	Primary hypertension		Renin inhibitors versus placebo	
**Diabetes**					
	Hemmingsen et al [36]	Type 1 diabetes mellitus		(Ultra-)long-acting insulin analogs versus neutral protamine Hagedorn insulin or another (ultra-)long-acting insulin analog	
	Gnesin et al [37]	Type 2 diabetes mellitus		Metformin monotherapy versus no intervention, behavior-changing interventions, or other glucose-lowering drugs	
**Dietary supplements**					
	Burckhardt et al [38]	Mild cognitive impairment or dementia due to Alzheimer’s disease		Souvenaid versus placebo	
	Showell et al [39]	Women who are subfertile		Supplementary oral antioxidants versus placebo, no treatment or standard treatment, or another antioxidant	

^a^Table S1 in Multimedia Appendix 1 summarizes the literature searches that we performed to select systematic reviews in each setting.

**Table 3:** Articles retrieved by the literature searches conducted in the published SR^a^ and numbers of articles available for analysis in this study^b^.

SR	PubMed-indexed articles retrieved in the SR, n	Source of articles ultimately used in the SR^c^			Dataset developed for this study			
		Any, n	PubMed^d^, n	Articles retrieved from PubMed by our search^e^, n		PubMed-cited articles included in the SR retrieved by our search, n (%)^f^	Total articles available for analysis using RMES^g,h^, n	Articles included in the SR, n (%)^f^
Zengerling et al [28]	849	42	16	368		16 (100)	335	16 (100)
Ferrara et al [29]	4935	31	10	5688		8 (80)	5612	8 (80)
Safi et al [30]	2471	56	53	2471		51 (96.2)	2184	44 (83)
Sethi et al [31]	3658	61	51	3779		47 (92.2)	3341	46 (90.2)
Roaldsen et al [32]	516	7	7	1411		5 (71.4)	1373	5 (71.4)
Martí-Carvajal et al [33]	359	10	7	494		7 (100)	489	7 (100)
Wang et al [34]	978	15	12	4153		11 (91.7)	4043	11 (91.7)
Musini et al [35]	1252	23	11	4656		11 (100)	4457	11 (100)
Hemmingsen et al [36]	2872	202	51	2924		50 (98)	2603	46 (90.2)
Gnesin et al [37]	3549	46	36	3684		7 (19.4)	3484	7 (19.4)
Burckhardt et al [38]	37	19	4	47		4 (100)	46	4 (100)
Showell et al [39]	865	79	47	992		40 (85.1)	973	40 (85.1)

^a^SR: systematic review.

^b^Tables S2A-S2L in Multimedia Appendix 1 show the search queries used for each SR. Table S3 in Multimedia Appendix 1 shows the filters applied for each review. Tables S4A-S4L in Multimedia Appendix 1 list the articles analyzed using RMES.

^c^Source of the articles used by the authors in the SR includes articles retrieved from PubMed, Embase, or other databases, and manual review of published reference lists, for example.

^d^Number of PubMed-indexed articles that were used by the authors in each SR. This number was used as the denominator to calculate percentages of articles included in the SR.

^e^Number of articles retrieved from PubMed using the modified search queries shown in Table S2A-S2L in Multimedia Appendix 1.

^f^The values in column “PubMed, n” are the denominators.

^g^RMES: Rapid Medical Evidence Synthesis.

^h^PubMed-indexed articles with an abstract.

### Development of the Dataset for RMES Screening

We applied the relevant search terms to develop datasets comprising PubMed-indexed articles for each systematic review (Table 3). The number of articles retrieved ranged from 47 to 5688 with or without an abstract, and from 46 to 5612 for articles with an abstract that could be analyzed using RMES. The articles retrieved from PubMed for each systematic review are listed in Tables S4A-S4L in Multimedia Appendix 1.

The authors of the systematic reviews generally searched MEDLINE through the Ovid portal, which provides researchers with more comprehensive search queries (including the ability to search for pairs of words that are adjacent or have one or more words in between) and filters than PubMed. We therefore decided to use broader search queries in order to maximize the articles retrieved and reduce the risk of inadvertently missing any references as a consequence of the initial search itself.

We then checked the articles and labeled those that were ultimately included in each systematic review. Our search retrieved most or all of the PubMed-indexed articles included by the authors. However, 1 exception was Gnesin et al [37], who included 36 PubMed-indexed articles, but only 7 of these were identified in our searches despite retrieving a comparable number of articles from PubMed (3684 vs 3549 in Gnesin et al [37]). However, we subsequently identified that the authors included some studies published outside the specified time period (2014-2019) in the search terms that were not retrieved by our recreated search.

### Accuracy of the RMES Filters

After preparing the dataset for article screening, the numbers of articles filtered by RMES, for all 4 filters, relative to the number of articles included in each systematic review were recorded. The data for each individual systematic review are presented in Table S4 in Multimedia Appendix 1. The selection success rates for each systematic review after applying all filters are presented in Table 4. The detailed performance data, including the selection reduction rates and numbers of articles included or excluded by the RMES filters, are reported in Table S5 in Multimedia Appendix 1.

Using filter 1, which comprised study type only, RMES correctly labeled 57.1% to 100% (median 80.9%) of the articles included in the reviews. This rate decreased slightly when we applied filter 2, which comprised study type + disease, ranging from 34.1% to 81.8% (median 65.2%). Using filter 3, comprising study type + intervention, the rate ranged from 0% to 100% (median 70.5%). Finally, when we applied filter 4, comprising study type + disease + intervention, the hit rate ranged from 0% to 81.8% (median 58.6%). The following sections show the results for 4 of the systematic reviews as representative examples with different levels of performance in terms of the reduction rate.

**Table 4:** Performance of the Rapid Medical Evidence Synthesis (RMES) filters: selection success rates^a^.

Systematic review	Filter 1: study type	Filter 2: study type + disease	Filter 3: study type + intervention	Filter 4: study type + disease + intervention
Zengerling et al [28] (n=16), n (%)	12 (75)	12 (75)	9 (56.3)	9 (56.3)
Ferrara et al [29] (n=8), n (%)	8 (100)	3 (37.5)	7 (87.5)	3 (37.5)
Safi et al [30] (n=44), n (%)	36 (81.8)	15 (34.1)	25 (56.8)	11 (25)
Sethi et al [31] (n=46), n (%)	40 (87)	30 (65.2)	37 (80.4)	28 (60.9)
Roaldsen et al [32] (n=5), n (%)	5 (100)	4 (80)	4 (80)	3 (60)
Martí-Carvajal et al [33] (n=7), n (%)	5 (71.4)	5 (71.4)	5 (71.4)	5 (71.4)
Wang et al [34] (n=11), n (%)	11 (100)	7 (63.6)	11 (100)	7 (63.6)
Musini et al [35] (n=11), n (%)	11 (100)	9 (81.8)	11 (100)	9 (81.8)
Hemmingsen et al [36] (n=46), n (%)	34 (73.9)	30 (65.2)	32 (69.5)	30 (65.2)
Gnesin et al [37] (n=7), n (%)	4 (57.1)	4 (57.1)	4 (57.1)	4 (57.1)
Burckhardt et al [38] (n=4), n (%)	3 (75)	3 (75)	0 (0)	0 (0)
Showell et al [39] (n=40), n (%)	32 (80)	14 (35)	15 (37.5)	6 (15)
Overall performance, median (range)	80.9% (57.1%-100%)	65.2% (34.1%-81.8%)	70.5% (0%-100%)	58.6% (0%-81.8%)

^a^Tables S2A-S2L in Multimedia Appendix 1 show the search queries used for each systematic review. Table S3 in Multimedia Appendix 1 shows the filters applied for each review. Tables S4A-S4L in Multimedia Appendix 1 list the articles analyzed using RMES. Table S5 in Multimedia Appendix 1 shows the number of articles excluded by each filter, number of articles selected by the filter (for manual screening), and the reduction rates.

#### Zengerling et al [28] (Systematic Review #1)

Zengerling et al [28] retrieved a total of 849 articles from PubMed, of which 16 were included in their systematic review, all of which had abstracts. A further 26 articles were obtained from other sources. We applied RMES to 335 articles retrieved from our own search of PubMed. Using filters 1-4, RMES reduced the number of articles for manual screening by 89.3% (299/335), 94% (315/335), 93.1% (312/335), and 95.5% (320/335), respectively. When we compared the articles retained by each filter relative to those included in the systematic review, the success rates were 75% (12/16), 75% (12/16), 56.3% (9/16), and 56.3% (9/16), respectively. Filters 1-4 excluded 4, 4, 7, and 7 articles, respectively, that had been included by the authors.

#### Wang et al [34] (Systematic Review #7)

Wang et al [34] initially retrieved 978 articles from PubMed, of which 12 were included in their systematic review, and a further 2 were obtained from other sources. Our search of PubMed retrieved 4153 articles, of which 4043 had abstracts. Among these, 11 were included in the original systematic review and had an abstract. Filter 1 reduced the number of articles to 656, corresponding to a reduction rate of 83.8% (3387/4043); but this included all 11 articles with a selection success rate of 100% (11/11). Filter 2 reduced the number of articles by 93.9% (3798/4043) and filter 3 reduced the number of articles by 88.6% (3584/4043); the corresponding success rates were 63.6% (7/11) and 100% (11/11). Finally, when we applied filter 4, the reduction rate was 95.5% (3860/4043) and the success rate was 63.6% (7/11).

#### Hemmingsen et al [36] (Systematic Review #9)

As a third example, Hemmingsen et al [36] conducted the largest systematic review, comprising 202 articles of which 51 were indexed in PubMed; the authors of that study screened 2872 PubMed-indexed articles. Our PubMed search retrieved 2924 articles (2603 with abstracts), of which 50 had been included by the authors (47 with abstracts). Using filters 1-4, RMES reduced the number of articles by 66% (1718/2603), 69.6% (1811/2603), 69.2% (1802/2603), and 72% (1873/2603), with selection success rates of 73.9% (34/46), 65.2% (30/46), 69.5% (32/46), and 65.2% (30/46), respectively. Filters 1-4 excluded 12, 16, 14, and 16 articles, respectively, that had been included by the authors.

#### Showell et al [39] (Systematic Review #12)

As a fourth example, Showell et al [39] retrieved 865 articles, of which 47 were indexed in PubMed. In our search, we retrieved 992 articles, of which 40, all with abstracts, were included by the authors in their systematic review. Filter 1 had a smaller reduction rate for this review (48.1%; 468/973) than for the other systematic reviews. The reduction rates using filters 2-4 were 84.9% (826/973), 87.5% (851/973), and 95.2% (926/973), respectively. Comparing the articles selected by the filters with the articles included in the systematic review, the success rates for filters 1-4 were 80% (32/40), 35% (14/40), 37.5% (15/40), and 15% (6/40), respectively. The latter value, in particular, was second-lowest for all reviews. The lowest was 0% (0/4) for the review by Burckhardt et al [38], for which only 4 articles were analyzable.

## Discussion

### Principal Findings

We used RMES to automatically screen the titles and abstracts of 46-5612 PubMed-indexed articles included in 12 systematic reviews across 6 medical fields. The number of PubMed-cited articles included in the reviews ranged from 4 to 47. We found that the median (range) percentage of articles correctly labeled by RMES using filters 1-4 were: 80.9% (57.1%-100%), 65.2% (34.1%-81.8%), 70.5% (0%-100%), and 58.6% (0%-81.8%), respectively. These results demonstrate the potential value of each filter for screening articles to be included in a systematic review.

#### Value of Automated Literature Tools in Literature Screening

Systematic reviews play a fundamental role in EBM, allowing stakeholders to make more robust conclusions on the basis of data from multiple clinical trials, larger sample sizes, and across different clinical settings (eg, countries, ethnicities, and clinical background) than is possible from a single RCT. The massive number of RCTs conducted over the last 20-30 years, in particular, can make it particularly difficult for stakeholders to retrieve relevant studies and publish EBM in a timely manner [2].

The initial screening of articles to be included in systematic reviews is a particularly laborious process with a significant workload. Semiautomated, AI-based software tools, such as RMES, have the potential to greatly reduce the resources and time taken to perform literature screening. RMES could facilitate literature screening in several settings, including screening of large numbers of articles or to accelerate the screening process. Although high accuracy is not provided with the current AI, this software could be used within a setting where a quick overview of the field is required with a limited research source. For example, it could be used for a feasibility check, or as a source of information for companies or researchers to make decisions about their future plans. With improvement in the discrimination accuracy, it may be possible to use this software as a substitute for the second or third reviewer in the future.

Using RMES and screening the articles using each filter, we found that filter 4, which comprised study type + disease + intervention, had the greatest impact on excluding potential articles with the highest reduction rates (ranging from 72% to 98.3%), whereas filter 1 had the lowest reduction rates (ranging from 48.1% to 93%). This is unsurprising considering that filter 4 is more restrictive since it combines study type with terms for the disease and the intervention, whereas filter 1 only screens for study type (ie, RCT). When comparing the success rates using the articles included in the published systematic reviews as a reference, filter 1 retained the highest proportion of articles with success rates of 100% for 4 systematic reviews (ranging from 57.1% to 100%). As we might expect, increasing the complexity of the filters had a corresponding effect on the success rates, which were lowest for filter 4, ranging from 0% to 81.8%. Intriguingly, filters 2 and 3 had varying impact on the reduction and success rates. This may reflect the complexity of the disease of interest or the treatments being evaluated, because multiple different phrases or terms might be used to refer to the specific disease or treatment, or the terminology does not map clearly to MeSH terms. This is particularly relevant to an AI model based on NLP, where certain terms or combinations of terms may not have been incorporated into the training. Overall, these results demonstrate the potential for using RMES to screen titles and abstracts through appropriate and careful use of the in-built filters. Nevertheless, some care is needed to consider the risk of excluding relevant articles when applying study type, disease, and intervention filters.

#### Usability of RMES in Literature Screening

There are some aspects of RMES that should be considered when incorporating this tool into literature screening. Manual screening is still required to verify the screened articles for systematic reviews; however, this reflects the situation where multiple reviewers usually screen the articles independently to reduce bias. RMES is currently limited to PubMed due to its free availability, whereas other databases may require subscriptions. Although PubMed is probably the most widely used database, systematic reviews that need to adhere to PRISMA (Preferred Reporting Items for Systematic Reviews and Meta-Analyses) or Cochrane Collaboration guidelines should also involve screening of articles from other databases, including Embase and clinical trial registries [43].

RMES can only perform screening of articles with a title and abstract. Some article types, such as brief reports and letters, often lack a published abstract. These article types should be left for the second round of screening because they may include data relevant to the systematic review [43,44]. Therefore, manual screening of such articles is required, regardless of whether the author uses RMES or performs the screening manually. For example, Hemmingsen et al [36] and Zengerling et al [28] both considered but excluded letters, after full-text review, from their systematic reviews. It is also important to carefully apply the filters to avoid excluding relevant articles.

We should also discuss possible reasons why the RCT classification was less efficient than might be expected. One possible factor is that there was an error in classifying RCTs that may be due to the limitation of tokens in PubMedBERT, such that the information required to classify a study as an RCT may not have been included within the sentences sent to PubMedBERT. Improvement in the section classification model may result in a better result within the limitation of the 512 tokens in PubMedBERT. This may reflect a fundamental problem when using key words and filters to retrieve articles from PubMed because not all RCTs are labeled as such, and other types of articles (eg, observational studies and reviews) may include terms that would result in them being mislabeled as RCTs. We note with interest that even the Screen4Me service developed by the Cochrane Center involves a combination of a machine learning algorithm and manual review by crowdsourcing to identify RCTs [16,24]. A recent study that examined the performance of this service yielded a specificity of 80.71%, indicating the difficulty of labeling articles as an RCT, even when performing manual review [16]. Considering these challenges, it is perhaps not surprising that some RCTs were excluded by RMES. Manual screening remains an important foundation for systematic reviews, and modern tools can be used to support this process.

As mentioned in the introduction, a number of programs and services have been developed to assist literature searches [3-24]. Although some studies have previously reported their performance, the results cannot be compared directly due to the use of different study designs, datasets, objectives, and metrics. In the future, it would be of interest to directly compare the performance of each program using a specific clinical setting.

### Study Limitations

Some limitations of this study warrant mention. In particular, we limited the systematic reviews to those that used RCTs, so we could not assess the accuracy of RMES for identifying other study types. Only articles indexed in PubMed were screened. The possible impact of RMES on reducing researcher time was not assessed. Furthermore, the study was conducted by nonspecialists, and we determined the accuracy of RMES by using the articles included by the authors of each review as a reference. We did not re-evaluate the validity of those articles or search for new articles relevant to the systematic review. Because we used RMES to screen the titles and abstracts (ie, the first screening round) relative to a published list of articles included in systematic reviews following manual screening of the full texts (ie, after the second screening round), and the systematic reviews did not publish the complete list of articles retrieved from the literature searches, we could not determine metrics such as recall or precision, or compare the accuracy of the replicated search.

### Conclusions

In conclusion, this study provides insight into the performance and capabilities of RMES for the initial screening of the titles and abstracts of articles included in previously published systematic reviews. RMES has the potential to reduce the workload involved in the initial screening of articles retrieved from PubMed when performing systematic literature reviews.

## Appendix

Multimedia Appendix 1 Supplementary tables.

## Abbreviations

AI: artificial intelligence
EBM: evidence-based medicine
MeSH: Medical Subject Headings
NLP: natural language processing
PICO: Participants, Intervention, Control, and Outcome
PRISMA: Preferred Reporting Items for Systematic Reviews and Meta-Analyses
RCT: randomized controlled trial
RMES: Rapid Medical Evidence Synthesis

## Data Availability

All data generated or analyzed during this study are included in this published article and its supplementary files.
